# Cellular morphological trait dataset for extant coccolithophores from the Atlantic Ocean

**DOI:** 10.1038/s41597-024-03544-1

**Published:** 2024-07-02

**Authors:** Rosie M. Sheward, Alex J. Poulton, Jeremy R. Young, Joost de Vries, Fanny M. Monteiro, Jens O. Herrle

**Affiliations:** 1 Institute for Geosciences, Goethe-University Frankfurt, Frankfurt am Main, Germany; 2 The Lyell Centre for Earth and Marine Science, Heriot-Watt University, Edinburgh, UK; 3 Department of Earth Sciences, University College London, London, UK; 4 BRIDGE, School of Geographical Sciences, University of Bristol, Bristol, UK; 5 Biodiversity and Climate Research Centre (BIK-F), Frankfurt am Main, Germany

**Keywords:** Carbon cycle, Biodiversity, Microbial biooceanography, Marine biology

## Abstract

Calcification and biomass production by planktonic marine organisms influences the global carbon cycle and fuels marine ecosystems. The major calcifying plankton group coccolithophores are highly diverse, comprising ca. 250–300 extant species. However, coccolithophore size (a key functional trait) and degree of calcification are poorly quantified, as most of our understanding of this group comes from a small number of species. We generated a novel reference dataset of coccolithophore morphological traits, including cell-specific data for coccosphere and cell size, coccolith size, number of coccoliths per cell, and cellular calcite content. This dataset includes observations from 1074 individual cells and represents 61 species from 25 genera spanning equatorial to temperate coccolithophore populations that were sampled during the Atlantic Meridional Transect (AMT) 14 cruise in 2004. This unique dataset can be used to explore relationships between morphological traits (cell size and cell calcite) and environmental conditions, investigate species-specific and community contributions to pelagic carbonate production, export and plankton biomass, and inform and validate coccolithophore representation in marine ecosystem and biogeochemical models.

## Background & Summary

Coccolithophores (Prymnesiophyceae) are abundant calcifying marine phytoplankton that are major contributors to the marine carbon cycle^1,2^ through their dual production of organic carbon (biomass) and biosynthesis of their inorganic carbon (calcium carbonate, calcite) exoskeletal plates. Coccolithophore calcification and photosynthesis are highly dynamic cellular processes that respond to changes in cell size, cell stoichiometry (elemental content), and the morphology and production rates of the calcite plates (coccoliths) that form the exoskeleton (coccosphere) of each cell^3,4^ that can occur as cell physiology responds to fluctuating environmental conditions. At the community level, changes in population growth rates and assemblage composition, including changes in the relative and absolute abundance of species present with different morphological traits and therefore degrees of calcification, influences the contribution of coccolithophores to pelagic carbonate production and carbonate flux to the deep ocean^5–7^. Variability in surface ocean carbonate production^2^, of which coccolithophores represent ca. 50–90%^1,8,9^, and carbonate export affects ocean alkalinity on shorter timescales and therefore the air-sea flux of carbon dioxide^9–12^. On longer timescales (Myr), the production and sedimentation of biogenic carbonate (the counter carbonate pump) acts as a long-term CO_2_ sink^13^ that helps to regulate global temperatures^14^. Given the major role of coccolithophores in these global processes and the sensitivity of coccolithophore physiology (including calcification) to environmental stressors^15–22^, understanding the biogeochemical and ecological consequences of the variability in coccolithophore calcification and productivity and the drivers of this variability have been pressing research questions for more than 40 years.

Coccolithophore morphological traits (i.e., cell size, coccolith size and morphological features, number and arrangement of coccoliths around the cell) are a principal determinant of coccolithophore calcification. There are approximately 250–300 (morpho)species of extant coccolithophores^23^ across two life cycle stages (diploid and haploid) that exhibit extensive morphological diversity^24^, which is critical for understanding the contribution of different coccolithophore species and coccolithophores as a functional group to marine carbon cycle dynamics. Despite this, coccolithophores are regularly regarded as a homogenous group and interspecies differences in size, calcification and physiological traits are often overlooked in common analyses. For example, coccolithophores are usually parameterised as a single ‘calcifying phytoplankton’ class in many Earth system models^25^. This is often unavoidable due to both analytical complexity and data availability, as most data for extant coccolithophore cellular biomass and calcite have been measured through the geochemical analysis of culture-derived material for the relatively small number of species that have been isolated^26^. The species *Emiliania huxleyi* has been the most extensively studied, with over 1000 experiments quantifying biomass and calcite content in this species from a range of genetic strains and under a range of environmental conditions^21^. Limited data for the organic and inorganic carbon content of other coccolithophore species are available for some species that are available in culture (e.g., *Gephyrocapsa*, *Coccolithus*, *Calcidiscus, Syracosphaera*, *Scyphosphaera*)^27–29^ but are based on a comparatively small number of studies that represent only a handful of species or strains.

Alternatively, morphometric traits can be used to estimate cellular-level calcite content for a wide diversity of extant coccolithophore species. A morphometric approach to calculating cellular calcite content is based on estimating the volume of individual coccoliths and the number of coccoliths per cell^30,31^. Morphometric-based calcite estimates generally agree well with calcite estimates derived through other approaches^32–35^ (see Technical Validation). With due consideration for the distinct morphological characteristics for each species, morphometric-based cellular calcite estimates can be made across the full diversity of extant and extinct species. The approach can be applied to coccoliths of any size or thickness and uses measurements taken on both entire, intact coccospheres and on individual coccoliths. Morphometric-based cellular calcite estimates are also truly cellular, as opposed to the assay-based measurements that average population calcite (including the calcite of dead cells and shed coccoliths) across population abundance and overestimate mean cell calcite as a result. Morphometric-based estimates of coccolith and cellular calcite content have been widely used to estimate pelagic carbonate production and carbonate export fluxes (sometimes using a species’ mean coccolith size and the number of coccoliths per cell only)^36–45^, and to quantify calcification responses to environmental and physiological change in both micropaleontological studies^46–48^ and laboratory experiments on *E. huxleyi*, *Coccolithus*, *Calcidiscus*, and *Helicosphaera*^4,49–52^. For the remaining species of extant coccolithophores, very little is known concerning their species- or genus-specific biomass and calcite.

Here, we present a novel dataset of cellular-level extant coccolithophore morphological traits^53^, including coccosphere and cell size, coccolith size, number of coccoliths per cell, and morphometric-based estimates of cellular calcite content. All data were measured from pelagic samples obtained from the Atlantic Ocean during the Atlantic Meridional Transect (AMT) cruise 14 (April-June 2004). The samples span several hydrographic provinces, including northern gyre, equatorial, subtropical gyre, and temperate waters^54,55^, thus capturing natural variations in morphology that arise due to genetic and phenotypic diversity. This dataset provides some of the first estimates of cellular calcite diversity across a broad diversity of extant coccolithophores from the Atlantic Ocean. The dataset presents 4712 morphometric measurements from 1074 individual coccospheres representing 25 extant coccolithophore genera and 61 species, representing all major families except for the Coccolithales and 80% of common genera identified during AMT 14^55^. The dataset offers reference data for taxon-specific size and calcification traits that can be used to investigate the contribution of coccolithophores to the global carbon cycle, spatial and temporal variability in coccolithophore calcite content, the links between morphometric traits (such as coccolith size) and the physiochemical properties of seawater, and trait responses to climate variability. The dataset, supporting data, and the archived images used to obtain the morphometric data are openly accessible through Zenodo^53,56,57^.

## Methods

### Field samples

Samples were collected during the Atlantic Meridional Transect 14 cruise (28 April to 1 June 2004). The cruise track ran from the Falkland Islands to the UK covering a sea surface temperature range of 5 to 27 °C and a dissolved nitrate range of 1 to >10 µmol N L^−1^ ^54,58^. The coccolithophore community during AMT-14 has previously been investigated over the euphotic zone at 19 stations^55^. For coccolithophore analysis, 1-2 L of a 20 L seawater sample collected using a rosette sampler from between 5 and 13 water depths (55, 33, 14, 1, and 0.1% of surface irradiance plus additional water depths at some stations) was gently filtered onto 25 mm polycarbonate filters with a 0.45 µm pore size. Filters were dried at room temperature and stored until imaging. For the purposes of this morphological trait dataset, a selection of samples were examined, covering a range of latitudes and water depths (Table 1) to ensure that morphometric data from a wide range of both common and less common extant coccolithophore species could be measured.

**Table 1:** Overview of AMT-14 CTD stations used for the extant coccolithophore morphological trait database.

Station	Hydrographic province^a^	Latitude	Longitude	Water depth (m)	Temp. (°C)	% surface irradiance	N (µM)	Chl (mg m^−3^)	Cells (mL^−1^)^b^	Data collected^c^	No. coccospheres^d^
CTD 6	TMP	−41.00	41.60	11	16.9	55	1.2	0.56	167.37	1	217
CTD 15	TMP	−33.00	31.00	15	22.1	55	<0.1	0.14	30.37	1	106
CTD 28	SG	−22.30	25.00	7	25.8	55	—	0.05	23.69	1	139
CTD 34	SG	−15.20	25.00	7	26.0	55	<0.1	0.06	20.02	1	73
				30	26.0	33	0.15	0.04	26.93	1	102
				59	26.0	14	<0.1	0.07	16.73	1	41
				90	25.3	4	<0.1	0.18	24.80	1	78
				120	23.5	1.5	–	0.18	31.47	1	58
				140	21.4	0.7	0.7	0.19	13.14	1	19
				158	20.2	0.4	1.8	0.17	10.30	1	27
				198	17.6	0.1	4.9	0.07	6.68	1	16
CTD 36	SG	−12.30	25.00	17	26.8	55	<0.1	0.05	23.55	1	63
CTD 44	EQ	−0.10	25.00	8	27.3	55	<0.1	0.17	31.08	1	56
CTD 41	EQ	−4.50	25.00	90	23.0	1	1.7	0.49	20.80	2	31; *Oolithotus*
CTD 71	NG	29.30	36.70	130	19.5	1	0.2	0.47	41.36	2	6; *Calciosolenia*
CTD 77	TMP	35.70	22.90	90	17.0	1	0.8	0.69	345.90	2	125; *Gephyrocapsa*

^a^Poulton *et al*.^55^, TMP = temperate, SG = southern gyre, NG = northern gyre, EQ = equator.

^b^Full assemblage count data is available for AMT-14 from the British Oceanographic Data Centre (www.bodc.ac.uk).

^c^Full assemblage coccosphere morphometric data were collected from six stations (enumerated as “1”). Additional morphometric data was collected for specific, less frequently encountered species from samples that were targeted based on available relative abundance counts (enumerated as “2”).

^d^Total number of measured intact coccospheres. If morphometric data was collected for a specific genus only, this is specified.

Coccolithophore morphometric data were collected from six stations at 55% surface irradiance (7 to 17 m water depth) and at a further seven water depths (30 m, 60 m, 90 m, 120 m, 140 m, 160 m and 200 m) at station CTD 34 in the southern subtropical gyre (Table 1). A further three samples identified as having higher abundances of generally less abundant taxa (*Oolithotus*, *Calciosolenia*, *Gephyrocapsa*) were also examined (Table 1).

### Imaging

Archived images from the study of Poulton *et al*.^55^ were re-examined here for the purposes of measuring key coccolith and coccosphere morphometric dimensions and estimating cellular calcite content. The sub-set of images examined for this study can be accessed through Zenodo^56^. The scanning electron microscopy (SEM) methodology applied to generate the images is described in Poulton *et al*.^55^ and follows Charalampopoulou *et al*.^59^. Segments of each filter were mounted on an aluminium stub and coated with gold. A SEM (LEO 1450VP Carl Zeiss combined with the software SmartSEM) at 5000x magnification was used to image each filter segment across 3-4 pre-defined meander-shaped, semi-continuous transects (zero overlap between fields of view). For each sample (station, water depth; Table 1), ca. 600–700 fields of view with a size 4.054 × 10^−3^ mm^2^ (1024 × 768 px, resolution of 13.92 px µm^−1^) were imaged, representing ca. 8–15 mL filtered seawater volume surveyed per sample. The number of intact coccospheres observed across these images varied from 16 to 217 between samples (Table 1).

### Extant coccolithophore morphometry

**Fig. 1:**
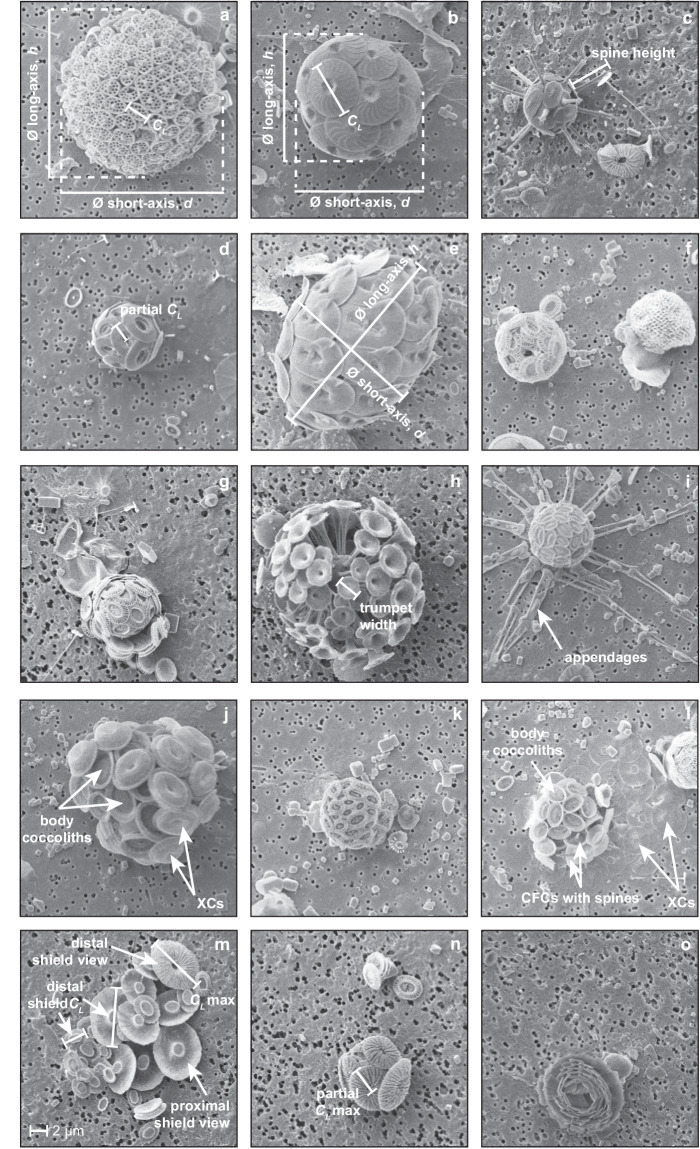
Illustrative examples of morphometric measurements and taxonomic specific morphological features. (**a**) Coccosphere of *Calyptrolithina multipora* (holococcolith-bearing; CTD34 60 m) illustrating the measurement of coccolith length (*C*_*L*_) and coccosphere diameter (∅) short-axis (*d*) and long-axis (*h*). (**b**) Same measurements illustrated as in (a) for a coccosphere of *Calcidiscus leptoporus* (CTD34 30 m). (**c**) Coccosphere of *Rhabdosphaera clavigera* var. *stylifera* (CTD15 13 m) illustrating the measurement of spine height. (**d**) Coccosphere of *Umbilicosphaera hulburtiana* (CTD36 17 m) showing the partial measurement of *C*_*L*_ between the centre point of the coccolith to distal shield rim edge, which is then multiplied by two in order to approximate *C*_*L*_ for specimens where the most flat-lying coccolith is partially overlapped by adjacent coccoliths. (**e**) Coccosphere of *Helicosphaera carteri* (CTD6 11 m) showing *h* and *d* measurements on a prolate spheroid coccosphere shape. (**f**) Coccospheres of *Emiliania huxleyi* (left) and the polycrater phase of *Alisphaera* spp. (right) illustrating very high numbers of very small coccoliths. (**g**) Coccosphere of *E. huxleyi* (CTD28 7 m) illustrating visible evidence of multi-layered coccospheres. (**h**) Coccosphere of *Discosphaera tubifera* (CTD28 7 m) illustrating the measurement of trumpet width used in the calculation of coccolith calcite for this taxon. (**i**) Coccosphere of *Michaelsarsia elegans* (CTD34 90 m) showing appendages. (**j**) Coccosphere of *Syracosphaera pulchra* (CTD34 30 m) showing the combination of body coccoliths and overlying exothecal coccoliths (XCs) on the same coccosphere. (**k**) Coccosphere of *Syracosphaera molischii* (CTD34 120 m). (**l**) Coccosphere of *Syracosphaera nodosa* (CTD34 120 m) showing circum-flagellar coccoliths (CFCs) with small spines and XCs. (**m**) A collapsed coccosphere of *Umbellosphaera tenuis* (CTD28 7 m) illustrating the wide range in coccolith sizes present on an individual coccosphere and distal shield measurements. (**n**) Intact *Umbellosphaera* coccosphere (CTD28 7 m) for comparison. (**o**) Coccosphere of *Florisphaera profunda* (CTD34 160 m) showing strongly overlapping layers of coccoliths. The 2 µm scale bar shown in (**m**) applies to all image panels.

For each sample, SEM images were surveyed sequentially until an intact coccosphere was observed. Each intact coccosphere was first identified to species level where possible (and genus level if species could not be identified) following the taxonomy of Nannotax3 (http://www.mikrotax.org/Nannotax3/) and supporting literature^23,60,61^ and forms a single data entry in the database comprising the following morphological measurements: coccolith length, coccosphere diameter, and number of coccoliths per cell. All measurements in the dataset therefore represent morphometric measurements obtained from intact coccospheres only, i.e., we do not make any measurements on loose, individual coccoliths and coccolith size data represents measurements made on coccoliths retaining their original arrangement within intact coccospheres. Measurements were made using the freeware ImageJ^62^ (v1.53a). Additional coccolith morphometrics were also measured when appropriate for each species, including coccolith width or the height of coccolith processes (e.g., spine height). The measurement procedure of these morphological features is described and discussed in detail below and an illustration of these measurements on example specimens from a range of species encountered within the samples is shown in Fig. 1. Further taxonomic considerations that influence the accurate collection of morphometric data are discussed in a following section.

#### Measurement of coccolith length, coccolith width and the size of species-specific morphological features

Coccolith length (*C*_*L*_) was measured on a single, flat-lying coccolith on the surface of the coccosphere where the outer rim of the coccolith distal shield was clearly visible (Fig. 1a,b). *C*_*L*_ measurements went precisely through the centre point of the coccolith. For species with larger central area processes (e.g., the spine of *Rhabdosphaera*, the trumpet-shaped processes of *Discosphaera*), the height of one process was also measured (Fig. 1c). For some coccospheres, a completely flat-lying coccolith was not visible on the upper coccosphere surface or the distal shield edge(s) of the most flat-lying coccolith were partially or fully concealed by overlapping adjacent coccoliths. In these cases, the length from central area mid-point to distal shield edge was measured and doubled to approximate coccolith length (Fig. 1d). Based on measurements of 100 loose coccoliths on a range of species, this method introduced a mean error of −0.6% (ranging ca. ±8%)^53^. Ideally, the measured flat-lying coccolith was representative of the size of all coccoliths forming the coccosphere. Coccolith width was similarly measured if necessary.

#### Measurement of coccosphere diameter

A wide range of coccosphere shapes exist across extant coccolithophores. Species are most commonly spherical or near spherical (60–75% of entries on Nannotax3^23^ are tagged as ‘equant’) but prolate spheroid and ellipsoid-shaped coccospheres are also relatively common (e.g., *Helicosphaera*, Fig. 1e) and ovoid to obpyriform, fusiform, tubular and bowl-shaped coccospheres are characteristic coccosphere shapes of certain species (for an overview of coccolith and coccosphere terminology, see Young *et al*.^63^). For easier comparison of coccosphere size across such a diversity of coccosphere shapes, we therefore recorded coccosphere diameter as equivalent spherical diameter, ∅, which is back calculated from coccosphere volume that has been calculated with a shape-specific equation for volume appropriate for the species in question. The size measurements required for the calculation of coccosphere volume (and equivalent spherical diameter) therefore depend on the shape of the coccosphere. As such, each genus or within-genus morpho-group has been assigned a best-fit mathematic shape label (Supplementary Table 1) that determines the volume calculation^64^ used to back calculate equivalent spherical diameter, as follows:

1 $${Prolate\; sphere}({PS}){volume}\,({\mu m}^{3})=\frac{\pi }{6}{d}^{2}h$$

2 $${Cone}+{half\; sphere}({CHS}){volume}\,({\mu m}^{3})=\frac{\pi }{4}h{d}^{2}$$

3 $${Double\; cone}({DC}){volume}\,({\mu m}^{3})=\frac{\pi }{12}h{d}^{2}$$

where *d* is the short-axis measurement (µm) and *h* is the long-axis measurement (µm) of the coccosphere (see also Fig. 1). Many spherical species (particularly those with placolith-type coccoliths) do not have precisely spherical coccosphere dimensions, as slight variability in coccosphere thickness can arise from the arrangement of overlapping coccoliths. As such, both long axis and short axis measurements are always made (Fig. 1a,b,e) and the volume of spherical, sub-spherical and prolate spheroid coccospheres (Supplementary Table 1) were all calculated using the equation for volume of a prolate sphere (Eq. 1). Although some coccosphere shapes are best described as ellipsoid, it was not possible to measure all axes necessary to calculate ellipsoid volume from two-dimensional SEM images, so volume was calculated as prolate spheroid (PS) for species with more elongated shapes (Supplementary Table 1).

#### Counting the number of coccoliths per cell

In SEM images, only one surface of the coccosphere is visible (Fig. 1). To count the number of coccoliths per cell, therefore, the number of coccoliths on the visible surface of the coccosphere was counted and this number was doubled to approximate the total number of coccoliths in the coccosphere (*C*_*N*_). Some species have coccospheres comprised of a very large number (>100) of very small (ca. 0.5 to 2 µm) coccoliths (e.g., the polycrater phase of *Alisphaera;* Fig. 1f). For these coccospheres, the number of coccoliths per cell was counted as accurately as possible but there is a degree of uncertainty in the counts (see Technical Validation).

### Morphometric-based estimates of cellular particulate inorganic carbon (PIC)

Morphometric-based estimates of cellular calcite (particulate inorganic carbon, PIC) depend on the number of coccoliths per cell and the PIC of each of those coccoliths. For each individual coccosphere, we first estimated coccolith PIC using the method of Young and Ziveri^30^ based on coccolith size (usually using coccolith length) and a shape factor related to coccolith cross-sectional shape, as follows:

4 $${coccolith\; PIC}\left({pg}{{CaCO}}_{3}\right)={{C}_{L}}^{3}\times {K}_{s}\times 2.7$$

Where *C*_*L*_ is coccolith size (µm), *Ks* is a species- or genus-specific shape factor, and 2.7 is the mass of calcite (pg µm^−3^).

Between species, *Ks* values vary by around an order of magnitude (minimum of ca. 0.01 to maximum of ca. 0.2 in extant species^30^). We used recommended species-specific *Ks* values from the literature^30,32,44,65,66^ or adapted the *Ks* values of a species with similar morphologies for species lacking a published *Ks* value. The specific coccolith length parameter used in the calculation (e.g., distal shield length or process height) and *Ks* value used for each genus or morpho-species group in the calculation of coccolith PIC are detailed in Supplementary Table 1. Cellular PIC was then calculated by multiplying coccolith PIC by the number of coccoliths per cell, as follows:

5 $${coccosphere\; calcite}\left({pg}\right)={coccolith\; PIC}\times {C}_{N}$$

Where *C*_*N*_ is the number of coccoliths per coccosphere/cell and coccolith PIC is calculated as in Eq. 4.

The relationship between coccolith morphology and coccolith calcite mass parameterised in Eq. 4, where cell-specific variability in coccolith calcite is driven principally by variability in coccolith size, represents a simplification of true cellular variation in coccolith calcite mass. Caveats and uncertainties related to this methodological approach, including variability in coccolith thickness, the use of species-specific shape factors, and measurement uncertainties, are discussed in detail in Young and Ziveri^30^ and in the Technical Validation.

### Inner cell size estimation

The organic biomass of phytoplankton cells is strongly related to cell volume^67^. In coccolithophores, the organic cell cytoplasm is covered by the calcite coccosphere so it is useful to report both coccosphere size and cell size dimensions. The inner coccosphere size (approximating the size of the organic cell) cannot be measured directly from SEM images, as the cell is obscured by the coccosphere (Fig. 1). We therefore estimated cell diameter as a function of coccosphere diameter, which can be precisely measured from SEM images (described above), using a species- or genus-specific function that estimates the percentage of coccosphere volume (from coccosphere diameter) that is occupied by cell volume, as follows:

6 $${Equivalent}({spherical}){cell\; diameter}(\mu m)=3\,\sqrt{\frac{6\left(\gamma \times \left(\frac{4}{3}\pi {\left(\frac{{\rm{\varnothing }}}{2}\right)}^{3}\right)\right)}{\pi }}$$

where ∅ is coccosphere diameter (µm) and *y* is the species- or genus-specific function relating coccosphere volume and cell volume (%). The percentage of coccosphere volume that is cell volume (*y*) is estimated using measurements of coccosphere diameter and cell diameter (as internal coccosphere diameter) from light microscopy images^4,68^ from multiple specimens using data from Sheward *et al*.^4,69^, Villiot *et al*.^29^ and newly collected data^57^. All values for *y* are reported in Supplementary Table 1. Estimates of cell size from coccosphere size are strongly influenced by coccolith thickness (or alternatively coccolith ‘height’ for spinose coccolith morphologies). For species with coccoliths possessing processes that extend a substantial distance from the cell (e.g., many Rhabdosphaeraceae species; Fig. 1c,h), values of *y* for estimating cell size from coccosphere size both including and excluding spine height are provided.

The majority of extant coccolithophore species have coccospheres formed of a single layer of abutting, overlapping and/or interlocking coccoliths. A notable exception to this is the species *Emiliania huxleyi*, which is well-known for producing coccospheres with multiple layers^70^. Other species that produce multilayer or pseudo-multilayer coccospheres include *Florisphaera profunda* and *Umbellosphaera tenuis*. We have accounted for the presence of (pseudo-)multi-layered coccospheres when converting from coccosphere size to cell size (see also details under *Emiliania*, *Florisphaera* and *Umbellosphaera* in the taxonomic-specific consideration below).

## Species- and genus-specific considerations

### Noelaerhabdaceae

#### Emiliania huxleyi

There are two additional factors influencing the calculation of cellular calcite in *Emiliania huxleyi*: variable degrees of coccolith calcite across the different morphotypes and the presence of multi-layered coccospheres.

Coccoliths of *E. huxleyi* have been categorised into informal morphotype groupings^23^ based on visual assessment of morphological features, including perceived degrees of calcification, e.g. ‘*E. huxleyi* Type A overcalcified’ or morphotypes defined as having open central areas (e.g., ‘*E. huxleyi* Type O’). Young and Ziveri^30^ have defined separate *Ks* values for ‘Type A group’ and ‘Type B group’ morphotypes of *E. huxleyi* and within *E. huxleyi*, Poulton *et al*.^40^ and Charalampopoulou *et al*.^43^ estimated from field biometric measurements on SEM images that variable coccolith morphologies led to a sixfold range in coccolith calcite. However, the calcite mass of coccoliths across different morphotypes and within morphotypes sharing the same morphological features are very variable^71–74^. Descriptive terms such as ‘*E. huxleyi* Type A overcalcified’ can therefore be misleading about differences in calcite mass between morphotypes, for example, a ‘Type A overcalcified’ coccolith with a closed or nearly closed central area can have less calcite mass than a “regular” ‘Type A’ coccolith^71^. For the purposes of our dataset, we did not attempt to classify *E. huxleyi* coccospheres into specific morphotypes or apply morphotype-specific shape factors in our calculation of coccolith calcite. In previous studies on the coccolithophore community composition of AMT-14^55^, relative abundance counts also did not distinguish between different *E. huxleyi* morphotypes so we cannot say which, if any, of the morphotypes is most abundant in our dataset. For simplicity, we have therefore used a single *Ks* value for *E. huxleyi* rather than different values for each morphotype, recognising that this will be an underestimate in some instance and an overestimate in others^72^ (estimated as coccolith calcite ±19%).

*E. huxleyi* often makes coccospheres consisting of more than one layer of coccoliths, with as many as 3–5 layers observed in culture^70,75,76^ and field samples^77–79^. This occurs especially under nutrient depleted conditions^68^, when cell division has slowed or ceased. For intact *E. huxleyi* coccospheres imaged using SEM, the presence of any additional coccolith layers is not always apparent, as the outermost coccolith layer may conceal underlying coccolith layers. Presence of multiple coccolith layers is often revealed when one or more of the coccoliths forming the outer coccolith layer becomes dislodged or the outermost coccolith layer has sheared off (Fig. 1g). Estimating cell diameter from the coccosphere diameter of multi-layered coccospheres by directly applying a fixed percentage conversion factor between coccosphere volume and cell volume (*y* = 86% for *E. huxleyi*; Supplementary Table 1) would introduce error, cell volume would be overestimated. For the purpose of estimating cell size using *y* for multilayer coccospheres of *E. huxleyi*, we have therefore artificially reduced the coccosphere diameter measurement to the diameter of an equivalent single-layer coccosphere using an estimate of mean *E. huxleyi* coccolith thickness. For two-layered coccospheres, we subtracted 2x coccolith thickness (0.13 µm x^2^ = 0.26 µm) from measured coccosphere diameter to simulate reducing the coccosphere diameter by the one extra layer of coccoliths present, for three-layered coccospheres we reduced coccosphere diameter by 4x coccolith thickness (0.13 µm x4 = 0.52 µm) to simulate reducing the coccosphere diameter by the two extra layers of coccoliths present, and so on. In SEM images, intact coccospheres of *E. huxleyi* sometimes have loose coccoliths lying directly next to the coccosphere (especially larger cells with multiple layers) and these were included in the *C*_*N*_ of the coccosphere to account for their calcite as they are presumably shed from the adjacent coccosphere.

Taxonomic note: In recent years, several publications have presented phylogenetic support for the inclusion of *E. huxleyi* in the genus *Gephyrocapsa*^80–82^ and *Gephyrocapsa huxleyi* is therefore increasingly used in publications. Here, we have continued to use the genus name *Emiliania* for *E. huxleyi* for three reasons^23^: (1) *E. huxleyi* and *Gephyrocapsa* have morphological distinctions that strongly influence their cellular calcite content, (2) distinguishing between *E. huxleyi* and *Gephyrocapsa* spp. is of practical use for biostratigraphic and paleoceanographic studies, and (3) *E. huxleyi* as a species name is widely recognised by non-experts so continued use of the genus *Emiliania* is useful for the wider communication of research outputs.

#### Gephyrocapsa

No specific considerations necessary.

### Coccolithaceae

No specific considerations were necessary for *Calcidiscus*, *Oolithotus*, or *Umbilicosphaera*.

### Calciosoleniaceae

#### Calciosolenia

This genus produces rhombic coccoliths. We are not aware of any previously published *Ks* value for *Calciosolenia* and so have back calculated a *Ks* value of 0.007 based on values for coccolith calcite (2.5 pg) and coccolith length (5 µm) published in Bollmann *et al*.^83^ based on circular polarizer birefringence-based measurements (n = 10) of coccolith thickness on specimens of Holocene age. Note that both the reported mass estimate and the coccolith length are likely to be underestimated, as the rim of *Calciosolenia* coccoliths is largely formed of vertically orientated crystal units (V-units) that are not birefringent under either cross polarised or circular polarised light. Coccospheres of *Calciosolenia* are strongly elongated and taper to a point at either end, giving them a shape that is best described mathematically as a ‘double cone’^64^. Measuring the dimensions of *Calciosolenia* coccospheres was sometimes challenging as the large coccospheres were often split across more than one SEM image. In these instances, if appropriate, we estimated the mid-point of the coccosphere within the image that contained the largest portion of the coccosphere and used 2x the measurement from this mid-point to the tip as the measure of length. For instances where the coccosphere was split over more than one SEM image, *C*_*N*_ was counted from all SEM images that contained parts of the coccosphere.

### Rhabdosphaeraceae

#### Acanthoica

Some coccoliths have well-developed spines, for example the polar coccoliths on *Acanthoica quattrospina*. The additional calcite content of these spinose coccoliths has not been explicitly accounted for in our calculations of cell PIC. Spine lengths are variable, ca. 6–17 µm in some specimens, and if we assume that the *Ks* of these spinose coccoliths is broadly similar to that of *Rhabdosphaera stylifera* (spine length for the calculation of calcite), the calcite of these spines would be ca. 9–136 pg, compared to a coccolith calcite of ca. 1 pg for regular body coccoliths (although this is likely an overestimate as *Acanthoica* spines have a less complex structure and are more tapered than *Rhabdosphaera*). The coccosphere calcite of *Acanthoica* specimens with spinose polar coccoliths is therefore underestimated. The average coccosphere thickness of *Acanothoica* is 0.7 µm and cell volume is on average 56% of coccosphere volume (n = 6) but can be smaller than this in some coccospheres (20–40% coccosphere volume).

#### Discosphaera tubifera

Coccolith calcite was calculated using trumpet width (Fig. 1h) following Young and Ziveri^30^ and *C*_*L*_ measurements refer to trumpet width in this taxon unless otherwise mentioned. Trumpet height was also measured so that coccosphere diameter including and excluding the trumpet height could be reported. Trumpet height can be variable across coccoliths on the same coccosphere (e.g., Fig. 1h; maximum trumpet height can be up to three-times longer than minimum trumpet height^84^). Occasionally, the trumpet-like processes of the coccoliths were broken off from the base plates.

#### Palusphaera and Rhabdosphaera xiphos

Coccospheres of *Palusphaera* and *R. xiphos* were difficult to distinguish at the resolution of the SEM images available. The primary distinguishing characteristic is that *Palusphaera* is monomorphic (all coccoliths have spines) but *Rhabdosphaera* is dimorphic (some but not all coccoliths have spines) (see taxonomic discussion in Archontikis and Young^61^). Coccospheres were distinguished where possible in the SEM images but were largely collapsed, which further inhibited confirmation of mono- or dimorphism and made accurate *C*_*L*_ and *C*_*N*_ measurements extremely challenging. As direct cellular morphometrics could not be taken accurately, *Palusphaera* and *Rhabdosphaera xiphos* were not included in the database.

#### Rhabdosphaera clavigera

Two varieties of *R. clavigera* are distinguished by how robust (var. *clavigera*) or delicate (var. *stylifera*) the spine structure is, although there is evidence of intergrading between the two forms^23,85^. Here, we separate the two varieties as the more robust spine has a greater calcite content (reflected in its higher *Ks* value) compared to the more delicate form. Note that, similarly to *Discosphaera*, there is variation in spine length ca. 5–10% between coccoliths on the same coccosphere^84^. *Rhabdosphaera* coccospheres are dimorphic, formed of coccoliths with and without spines (Fig. 1c). For the calculation of coccosphere calcite, it was assumed that 50% of the coccoliths were without spines (coccolith calcite for these coccoliths was calculated using *C*_*L*_ and a *Ks* value of 0.025, modified from *Acanthoica*^30^) and 50% of the coccoliths were with spines (calcite was calculated using spine length and a fixed value of 5 µm was used if spine length could not be measured). It was usually possible to accurately measure coccosphere diameter both including and excluding spine length and coccosphere size is reported as excluding spine length unless otherwise stated.

### Syracosphaeraceae

#### Calciopappus

*Calciopappus* coccospheres were extremely rare in the samples investigated and were not observed as intact coccospheres. We therefore do not report any data for *Calciopappus*.

#### Michaelsarsia

*Michaelsarsia* coccospheres were rare in our samples (n = 5). Each coccosphere typically has ca. 8–12 appendages each formed of 3–4 coccoliths^86^ (Fig. 1i). To avoid underestimating *Michaelsarsia* coccosphere calcite by omitting the calcite in these appendages, we assigned a value of 12.5 pg calcite per appendage (informed by a typical length of 5.5 µm for each appendage segment, a *Ks* value of 0.007 and assuming 4 segments per appendage). If the number of appendages is unknown, we assumed that 8 appendages were present (i.e., an addition of 100 pg calcite). This additional calcite was then added to the calcite of the main body coccoliths, which were calculated using a *Ks* value adapted from *Syracosphaera*.

#### Ophiaster

The appendage coccoliths of *Ophiaster* are also elongated but with a more solid structure than those of *Michaelsarsia* and each coccosphere usually has 4–7 long ‘arms’ of these osteoliths^86^. As the appendages of *Ophiaster* are longer and with a much greater number of component osteoliths, an approximation of the additional calcite represented by the appendages was estimated as follows: assuming that each of the osteoliths has the same *Ks* value as a *Florisphaera profunda* nannolith (*Ks* = 0.03^32^ based on the broadly similar shape) and each ‘arm’ contains on average 14 nannoliths of ca. 1.8 µm length, this would equate to 0.47 pg calcite per nannolith, 6.6 pg calcite per ‘arm’ and 33 pg additional calcite per coccosphere in total (assuming on average 5 arms). This fixed estimate of an additional 33 pg calcite for appendages was applied where appendages were clearly present (as many specimens are without appendages^86^). This estimate of appendage calcite is broadly comparable to the calcite contained in the body coccoliths of many specimens (i.e., including a calcite estimate for the appendages approximately doubles cell calcite content calculated on body coccoliths alone).

#### Syracosphaera

The genus *Syracosphaera* is very diverse^87^ (ca. 40 currently recognised species^23^). There is substantial morphological diversity in this genus, including coccolith size variability, different degrees of calcification, and a range of central area structures. However, four sub-groups of species with similar morphological characteristics can be distinguished (Nannotax3^23^ following Cros^88^, Young *et al*.^60^ and Kleijne and Cros^87^) and we used these morpho-groups as a starting point to simplify the choice of shape factor for calculation of cellular calcite content in this genus. In addition, many *Syracosphaera* species have circum-flagella coccoliths (CFCs) with relatively small spines and exothecal coccoliths (XCs) that form a partial or complete outer layer to the coccosphere (Fig. 1j). This range of features make it impractical to determine new species-specific *Ks* values for the calculation of coccolith calcite for *Syracosphaera*. For the calculation of cellular calcite, we therefore proceeded as follows:

- For *Syracosphaera* species that have a well-developed inner wall cycle and robust central area structures such as a boss or spine, we use the published *Ks* value of Young and Ziveri^30^ for *Syracosphaera pulchra* (*Ks* = 0.03). This includes species in the ‘*pulchra* group’ and *Syracosphaera mediterranea*, which has a thick rim cycle and low central mound. For this morpho-group (e.g., Fig. 1j), cell volume was assumed to be 65% of coccosphere volume (ranging between ca. 60 and 80% for species in the group).
- For species without either of these features we use the *Ks* values of Young and Ziveri^30^ for a ‘small’ *Syracosphaera* species (*Ks* = 0.015), even though the coccoliths in question may not be ‘small’. This includes ‘*borealis* type’ species, ‘*nodosa* group’ species (e.g., Fig. 1l), and *Syracosphaera maxima*. For this morpho-group, cell volume was assumed to be 75% of coccosphere volume.
- For species that have well-developed rims but no central area bosses or spines, we chose a relatively arbitrary ‘intermediate’ value of *Ks* = 0.022. This includes species in the ‘*molischii* group’ (other than ‘*borealis* type’ species; e.g., Fig. 1k). For this morpho-group, cell volume was assumed to be 75% of coccosphere volume.
- For coccospheres that could not be confidently identified to species level, a *Ks* value of 0.02 was used to calculate coccosphere calcite.
- The morphology of CFCs is often similar to that of the body coccoliths but with relatively small spines (Fig. 1l). We therefore used the same *Ks* value for CFCs as for body coccoliths.
- There is a much broader morphological diversity across XCs, which makes it challenging to apply any ‘one size fits all’ approach to account for differences in the calcite content of XCs in the calculation of cellular calcite across all *Syracosphaera* species. The morphologies of XCs have previously been tentatively classified into 12 groups^88^ spanning disk-like coccoliths (e.g., *Syracosphaera anthos*, *Syracosphaera nodosa*, *Syracosphaera lamina*, *Syracosphaera nana*, *Syracosphaera bannockii*), undulating coccoliths that have a distinct rim and central area (e.g., *Syracosphaera borealis*, *Syracosphaera molischii*, *Syracosphaera ossa*), dome-like/vaulted coccoliths (e.g., *Syracosphaera pulchra*), and caneoliths with a murolith morphology (e.g., *Syracosphaera dilatata*, *Syracosphaera prolongata*, *Syracosphaera noroitica*). As XCs can be numerous (e.g., Fig. 1j,l), we attempted to account broadly for the calcite of these XCs when present by counting the number of XCs present and using a *Ks* value of 0.02 for all morphologies to calculate the additional calcite contained in these coccoliths, recognising that this *Ks* value is an underestimate in some instances and an overestimate in others.
- An additional challenge presented by the presence of XCs is that they partially or sometimes entirely obscure the visibility of body coccoliths (Fig. 1j). In these instances, a best estimate of *C*_*N*_ was determined by either scaling up the number of body coccoliths visible over an unobscured portion of the cell, doubling the *C*_*N*_ of visible XCs when the size of XCs and body coccoliths are broadly comparable and the entire coccosphere is covered in XCs, or estimating *C*_*N*_ based on the range of *C*_*N*_ typical for the species (this is noted in the raw data file when applied).
- Many species of *Syracosphaera* are known to have holococcolith stages. See the remarks on ‘Holococcolithophores’ for further details.

### Helicosphaeraceae

No specific considerations were necessary for *Helicosphaera*.

### Alisphaeraceae

#### *Alisphaera* and *Alisphaera* polycrater phase (POL)

*Alisphaera* is a genus with a very high *C*_*N*_ per coccosphere and small coccoliths, especially in polycrater phase (Fig. 1f). Where *C*_*N*_ could not be accurately counted and/or *C*_*L*_ could not be accurately measured (particularly for polycrater phase coccospheres) from SEM images, the *C*_*N*_ /*C*_*L*_ range of the species reported on Nannotax3^23^ was used to guide our choice of a fixed *C*_*N*_ of 400 and fixed *C*_*L*_ of 0.8 µm for the calculation of cellular calcite.

### Umbellosphaeraceae

#### Umbellosphaera

*Umbellosphaera* coccoliths have umbelliform morphology, reminiscent of a flaring trumpet shape. Coccoliths forming the coccosphere have a range of distal shield sizes (as seen easily from collapsed coccospheres; Fig. 1m) that leads *Umbellosphaera* coccospheres to have a pseudo-multilayered structure that is reminiscent of a rainforest canopy structure, where the distal shields of the largest coccoliths overlay the distal shields of smaller coccoliths (Fig. 1n) whilst the proximal shields sit adjacent on the cell surface. This presents challenges for counting *C*_*N*_ accurately and accurately measuring a ‘representative’ coccolith size in *Umbellosphaera*. For *C*_*N*_, we therefore multiplied counted coccoliths by three (rather than by two for regular, single-layer coccospheres) to better account for the ‘hidden’ layer of coccoliths underneath the visible outer layer. For intact coccospheres, *C*_*L*_ and *C*_*W*_ of the largest coccolith distal shield were measured.

For the coccolith size measure used in the calculation of cellular calcite, we also adjusted the maximum (measurable) distal shield length to approximate an ‘average’ distal shield length that accommodated the variable lengths of *Umbellosphaera* coccoliths that occur on the same coccosphere. For 20 collapsed *Umbellosphaera* coccospheres (e.g., Fig. 1m), we measured the distal shield length of every coccolith (*C*_*N*_ of the collapsed coccospheres ranged from 10 to 43) and calculated coccolith calcite and coccosphere calcite^53^ using the *Ks* values of Young and Ziveri^30^ for *Umbellosphaera tenuis* and *Umbellosphaera irregularis* as appropriate, following Eq. 4 and Eq. 5.

We then back-calculated an ‘average’ *C*_*L*_ corresponding to the calculated cellular calcite and *C*_*N*_ of the cell and determined what percentage of the largest measured coccolith size (*C*_*L*_ max; Fig. 1m) this ‘average’ coccolith represented^53^, as follows:

7 $$ \mbox{`} {average}\mbox{'}{C}_{L}({\mu m}^{3})=3\,\sqrt{\frac{{coccosphere\; calcite}}{{K}_{S}{\times C}_{N}\times 2.7}}$$

For the 20 collapsed coccospheres measured, the ‘average’ coccolith size ranged between 55 and 81% of the largest measured coccolith^53^. For the calculation of *Umbellosphaera* cellular calcite, we therefore decided to use a *C*_*L*_ value of 0.7*C_*L*_ max, where *C*_*L*_ max is the coccolith length of the largest (measurable) coccolith on the intact coccosphere (e.g., Fig. 1n).

### Holococcolithophores and Nannoliths

#### Holococcolithophores

Coccolithophores have a haplo-diplontic life cycle, and typically produce heterococcoliths in the diploid life cycle stage and holococcoliths (HOL) during the haploid life cycle stage (e.g., Fig. 1a). Holococcoliths are formed from small, simple rhombohedral crystals and are morphologically distinct from heterococcoliths^89^. Holococcoliths do, however, occur in a range of different shapes and forms^60^. The calcite content of individual holococcoliths is currently unknown and we are not aware of any published values for inorganic carbon per cell measured from cultured holococcolith-bearing strains from which coccolith calcite and therefore *Ks* could be estimated. Here, we therefore used an estimated *Ks* value of 0.036 for all holococcolith-bearing coccospheres^45^.

If it was not possible to directly count *C*_*N*_ (as holococcoliths are often very small and coccospheres have high *C*_*N*_), *C*_*N*_ was estimated by back-calculating *C*_*N*_ using the surface area of the coccospheres and the surface area of the measured coccolith or an average *C*_*N*_ of that species was used if available from another published source. We assumed that cell volume is 80% of coccosphere volume for all holococcolithophore coccospheres.

### Nannolith-bearing taxa

#### Ceratolithus

*Ceratolithus* produces three different lith morphologies depending on the life cycle stage, including distinctive horseshoe-shaped nannoliths (ceratoliths, CER). No intact *Ceratolithus* coccospheres were observed in any of our samples so they are not present in the database.

#### Florisphaera profunda

The sheet-like, angular nannoliths of *Florisphaera* are arranged to form a coccosphere of strongly overlapping layers reminiscent of a globe artichoke (Fig. 1o). As a result, the coccosphere is very thick and, on SEM, it is not usually possible to accurately determine how many layers of coccoliths are present. Light microscopy and SEM images indicate that ca. 6 to 12 layers of overlapping coccoliths can be present^23,60,84^ (Fig. 1o) and that coccospheres may have up to ca. 220 nannoliths^84,90^. New LM measurements of corresponding coccosphere and internal cell diameter measurements from 20 *Florisphaera* coccospheres^57^ indicate that cell size is typically 3–5 µm and cell volume is ca. 10% of coccosphere volume (ranging from 3% to 21%)^57^. We therefore estimate cell size from coccosphere size assuming that cell volume is 10% of coccosphere volume. As precise estimates of *C*_*N*_ were not attainable, we assumed that *Florisphaera* coccospheres have a fixed *C*_*N*_ of 145, broadly in line with the average *C*_*N*_ reported in the literature for coccospheres for *Florisphaera profunda* var. *elongata* and *F. profunda* var. *profunda*^84^. The use of a fixed *C*_*N*_ obviously introduces significant uncertainty in the estimates of *Florisphaera profunda* cellular calcite (assuming a *C*_*L*_ of 2 µm and that the *C*_*N*_ of *Florisphaera* typically ranges between ca. 90 and 170, the use of the fixed *C*_*N*_ introduces an uncertainty of ca. 20–40%).

## Data Records

The extant coccolithophore morphometric trait dataset described in this study can be freely downloaded at zenodo^53^. This data record contains the main dataset “*1. Cellular morphometric trait dataset for extant coccolithophores from the Atlantic Ocean*” (Microsoft Excel file) and four supporting files: “*2. Descriptor – Cellular morphometric trait dataset for extant coccolithophores from the Atlantic Ocean*” (Microsoft Word file), “*3. Coccolith size, coccolith calcite and cellular calcite in collapsed Umbellosphaera coccospheres*” (Microsoft Excel file), “*4. Sensitivity of cellular calcite content to uncertainties in coccolith size, number of coccoliths per cell and shape factors*” (Microsoft Excel file), and “*5. Partial coccolith length and coccolith length measurements*” (Microsoft Excel file). This section describes the content of these files.

The *Cellular morphometric trait dataset for extant coccolithophores from the Atlantic Ocean* is formatted as a column-orientated table in Microsoft Excel format. Each row of the dataset represents the raw morphometric data (number of coccoliths per cell, coccolith length, coccolith width, coccosphere diameter long axis, coccosphere diameter short axis) and calculated morphological trait data (coccosphere volume, equivalent spherical coccosphere diameter, cellular calcite) obtained from a single intact coccosphere. Each entry includes ancillary information, including sample identifier (station and water depth), SEM image number and taxonomic classification (family, genus, species). A column entitled ‘Additional information’ records any additional details relevant to the accuracy of the measurements and calculated parameters, for instance stating the presence and amount of XCs additionally included in the cellular calcite calculation or the presence of a multi-layered coccosphere where this has been accounted for in the estimation of cell size from coccosphere size.

The contents of the four supporting files associated with the *Cellular morphometric trait dataset*^53^ are as follows:

File 2. *Descriptor* describes the column headings contained in the *Cellular morphometric trait dataset*.

File 3. *Coccolith size, coccolith calcite and cellular calcite in collapsed Umbellosphaera coccospheres* contains the coccolith size and coccolith calcite data measured from collapsed coccospheres of *Umbellosphaera* used to derive the adjustment of coccolith size used in the calculation of cellular calcite *in Umbellosphaera*.

File 4. *Sensitivity of cellular calcite content to uncertainties in coccolith size, number of coccoliths per cell and shape factors* contains the data used to perform the sensitivity analysis of cellular calcite to variability in morphometric parameters (see Technical Validation).

File 5. *Partial coccolith length and coccolith length measurements* contains coccolith measurements used to calculate the measurement uncertainty associated with measuring partial *C*_*L*_ on coccoliths where the rim of the distal shield is partly obscured by an overlapping coccolith.

In addition to this data record containing the *Cellular morphometric trait dataset for extant coccolithophores from the Atlantic Ocean* dataset and supporting files^53^, two new further records^56,57^ associated with the dataset are available:

- The photo record “*Atlantic Meridional Transect (AMT) 14: scanning electron microscopy images of the coccolithophore community*” is freely available for download at zenodo^56^ and contains the original SEM image files as tagged image file format (.tif) files. The identifier of each image file corresponds to the image number in the dataset *Cellular morphometric trait dataset for extant coccolithophores from the Atlantic Ocean*^53^.
- The dataset “*Extant coccolithophore cell size measurements*” (Microscoft Excel file) is freely available for download at zenodo^57^ and contains newly collected measurements of coccosphere diameter and cell diameter on individual coccospheres used to derive species- and genus-specific values for the percentage of coccosphere volume that is cell volume (*y*).

An overview table of the genus-specific morphometric parameters used to estimate cellular calcite (e.g. taxon-specific shape factors, size measurement used) and the range of estimated taxon-specific cellular calcite content that summarise the data contained in the *Cellular morphometric trait dataset for extant coccolithophores from the Atlantic Ocean*^53^ is available as Supplementary Table 1.

## Technical Validation

### Taxonomic coverage of the cellular morphometric trait dataset

**Fig. 2:**
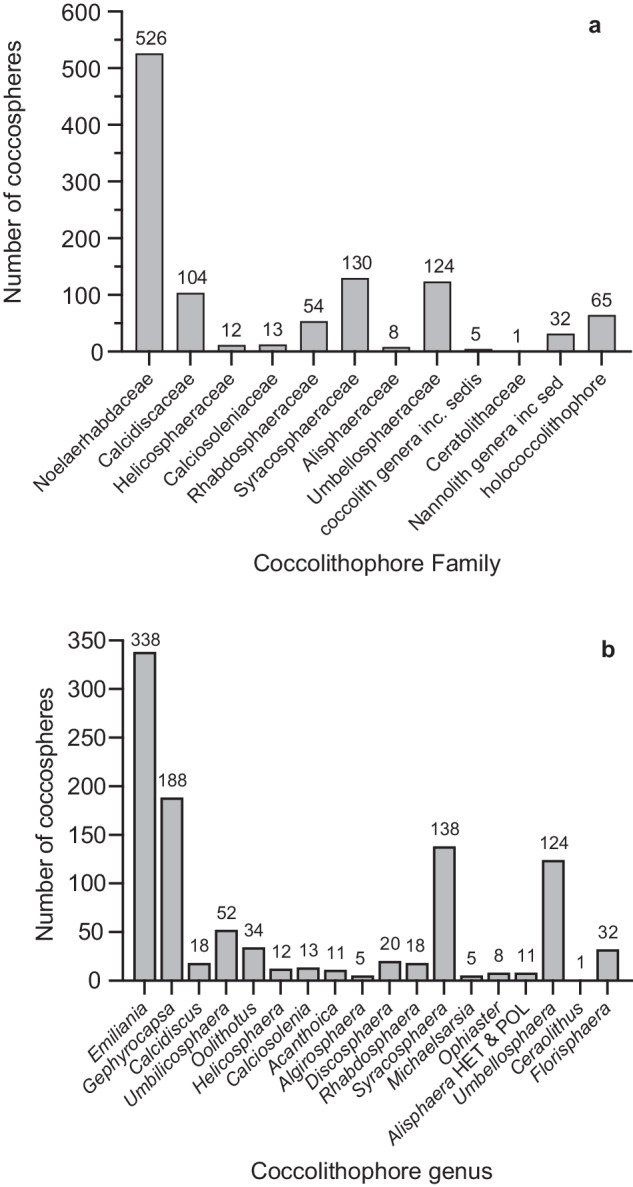
Taxonomic coverage of the morphometric dataset. (**a**) Coverage by coccolithophore Family. (**b**) Coverage by coccolithophore genus. Number of coccospheres in the morphometric dataset are labelled above each bar.

In total, 4712 morphometric measurements were measured on 1074 individual coccospheres representing 61 species from 25 genera (of the 31 most common genera that collectively constituted >95% total cell numbers during AMT 14^55^) (Fig. 2). The number of measurements for each genus represented in the dataset largely reflects the relative abundance of different species in the samples examined, as well as the structural stability of different coccosphere architectures (as some species coccospheres have a greater tendency to collapse, even under gentle filter pressure, and were therefore not measured).

Five genera (20%) have more than 50 coccospheres in the dataset and 14 genera (70%) have 10 or more coccospheres in the dataset (Fig. 2b). Some extant taxa are not present in our dataset for the following reasons: (1) the species was not observed because it is generally not present in the temperate to equatorial latitudes of the oceanic Atlantic ocean sampled during AMT-14 (e.g., *Coccolithus pelagicus* that has a biogeographic distribution in the northern hemisphere high latitudes, the genus *Braarudosphaera* that is known to thrive in hyposaline conditions, the predominantly polar Family Papposphaeraceae, and the neritic genera in the Families Hymenomonadaceae and Pleurochrysidaceae); (2) the species is generally rare (e.g., *Calyptrosphaera*, *Navilithus*, *Placorhombus*, and *Turrilithus*) or rare in AMT samples (140 of 171 species, or 82%, recorded during AMT-14 constituted <5% total cell numbers and are defined as rare^55^); (3) The species was not observed as intact coccospheres in our samples because it has a structural susceptibility to collapse and therefore could not be accurately measured (e.g., the deep photic species *Gladiolithus*). Additionally, we did not observe any *Scyphosphaera* or *Pontosphaera* coccospheres (intact or collapsed) in any of the samples we examined for this study. *Scyphosphaera apsteinii* was only recorded in two samples during AMT-14 and no species of the genus *Pontosphaera* were reported in any samples^55^.

### Size measurement errors using microscopy (SEM and LM)

SEM has a theoretical resolution of ca. 0.003 to 0.05 µm dependent on the electron source, the accelerating voltage, and the working distance. The optical resolution of LM is dependent on the objective used, the numerical aperture of the objective and the use of any immersion liquid. Coccolithophore researchers typically use a 100x/1.30 oil-immersion objective, which has an optical resolution of 0.26 µm. Combined with the pixel resolution of the SEM images, all *C*_*L*_ and ∅ measurements^53^ therefore have a maximum error of ca. ±0.12 µm. The LM measurements of coccosphere and cell size used to calculate the percentage of coccosphere volume that is cell volume (*y*)^57^ should be assumed to have a minimum error of ±0.26 µm. Note that the new size measurements taken here from SEM images^53^ and from LM images^57^ were not specifically controlled with a calibration standard (e.g., calibration microbeads)^91,92^.

### Comparison of morphometric-based estimates of coccolith calcite with other estimation methods

Due to the very small size of coccoliths (ca. 1 to 20 µm) and coccospheres (ca. 3 to 40 µm), the mass of individual coccoliths and coccospheres cannot be measured directly and so must be estimated using indirect approaches. There are several established methods used to estimate the calcite mass of coccoliths and/or coccospheres, each with advantages and limitations that also depend on the characteristics of the material being investigated (e.g., whether samples from culturing, plankton or sediment are used and whether coccolith calcite or coccosphere calcite is being measured). An extensive discussion of the advantages and limitations of each method is beyond the scope of our study. We refer readers to the literature cited in the following paragraphs for specific technical developments, particularly regarding birefringence-based methods for the quantification of coccolith calcite mass. Instead, we briefly summarise the main advantages and disadvantages of alternative methods for estimating coccolith and cellular calcite content within the scope of comparing our estimated cellular calcite data with data derived from other methodologies.

Estimates of coccolith calcite based on morphological measurements^30,93^, as used here, can be performed using measurements from either light microscopy images (LM) or scanning electron microscopy (SEM), as here. The high resolution of SEM images can resolve very small and lightly calcified details of coccoliths, often improving taxonomic classification of material relative to LM and enabling high accuracy morphometric data to be collected across a wide range of coccolith sizes and morphologies. A disadvantage of SEM is that the two-dimensional nature of the images allows only one hemisphere of the coccosphere to be viewed directly (Fig. 1) and therefore there must be a degree of estimation in the total number of coccoliths per cell. This is an advantage of LM, where the focal depth can be adjusted to observe and count each coccolith in the coccosphere^4,66,68^.

Alternatives to morphometric-based calcite estimates include birefringence-based mass estimates^83,94,95^, which are often used for calcite mass estimates of individual coccoliths. Technical advances in the calibration of birefringence-based methods for coccolith mass estimation have been made in recent years^32,83,96–99^, notably using circular polarized light rather than cross-polarised light and calibrating the calcite interference colours to the Michel-Lévy chart with additional technical management of the microscope, light source and imaging settings^32,83^. Despite these technical advances, mass estimates based on birefringence are limited by both the orientation of the crystal(s) in the coccolith and by coccolith thickness, which does have a detection limit: thickness limits imposed by optical physics are 1.34 µm^32,83^ to a maximum of 1.58 µm^32^ after which greyscale values repeat themselves. Thickness limits as high as 1.70 µm have also been published^99^. Birefringence-based methods work well for species with smaller or thinner coccoliths, such as *E. huxleyi*, but underestimate the calcite mass of coccoliths that exceed thicknesses of ca. 1.4 µm (e.g., larger specimens of *Coccolithus*, *Helicosphaera* and *Calcidiscus*)^83^. In addition to coccolith thickness limits, birefringence-based approaches systematically underestimate coccolith calcite mass in species where some or all of the coccolith is formed of vertically-arranged crystal units (V-units) that are not birefringent under either cross polarised or circular polarised light (e.g., *Coccolithus* spp., *Calcidiscus* spp., *Umbilicosphaera* spp.)^65,83,94,99^. Birefringence is also unsuitable for coccolith mass estimation when the coccolith is part of an intact coccosphere (as measured here), where overlapping coccoliths can increase the calcite brightness leading to significant calcite overestimation^100^.

Another commonly used approach in culturing studies is the analytical determination of mean calcite per cell through assays for total sample particulate carbon and particulate organic carbon (from an acidified sample), where total sample calcite is the difference between the two values (the inorganic carbon) and cellular calcite is determined by dividing the value by the number of cells present on the filter (when cells per millilitre and number of millilitres filtered are known). This approach does not capture intraspecific variability in cellular calcite and has the additional disadvantage that any ‘additional calcite’, e.g. from dead cells or shed coccoliths, is also averaged into the cellular calcite value and this can lead to the overestimation of mean cellular calcite^52^. Assay-derived cellular calcite estimates are effectively restricted to *E. huxleyi*, *Gephyrocapsa* spp., *Coccolithus* spp., *Calcidiscus* spp., *Syracosphaera pulchra* and *Helicosphaera carteri*, i.e., species that have been used in monospecific culturing experiments^27^. It is not possible to use assay-based analysis for the determination of taxon-specific cellular calcite content from a mixed population sample (i.e., a plankton sample from the field), as the nature of this analysis will produce a meaningless average value spanning all cells, regardless of their taxonomic affinity. This method is, however, well suited to estimating total organic and inorganic carbon content of a population.

Morphometric-based calcite estimates have been shown to compare well to estimates of coccolith calcite derived using other methodologies. Birefringence-based estimates of coccolith calcite are consistently in good agreement with morphometric-based estimates of coccolith calcite for species within the coccolith size and thickness range for which this method is optimised, especially when samples sizes are large enough to account for intraspecific variability^65,83,94^. In one study^32^, the calcite mass of coccoliths measured using both the same morphometric-based method as here and a birefringence-based method on the same samples showed good agreement in Noelaerhabdaceae and Umbellosphaeraceae coccoliths. For *E. huxleyi* and small placolith coccoliths, mean calcite mass based on *C*_*L*_ and the recommended Ks value of 0.02^30^ was 1.7 pg *versus* 2.2 pg derived through a birefringence-based method^32^. For *Umbellosphaera* spp. coccoliths, the morphometric-based estimate of mean coccolith calcite mass was 7.1 pg compared to a birefringence-based estimate of 7.5 pg^32^. A recent direct comparison study^33^ also showed that coccolith mass estimates for four strains of *E. huxleyi* obtained from morphometric data using SEM (as here) agreed well with estimates obtained through birefringence measurements of coccolith thickness using bidirectional circular polarisation^97,99^ with LM (mean calcite mass of Type A coccoliths was 2.09 pg based on morphometrics *versus* 2.02 pg based on birefringence, mean calcite mass of Type BC coccoliths was 1.71 pg based on morphometrics *versus* 1.82 pg based on birefringence; differences were not significant)^33^. For species in the orders Coccolithales (e.g., *Coccolithus, Calcidiscus*, *Umbilicosphaera*) and Zygodiscales (e.g., *Helicosphaera*), Linge Johnsen and Bollmann^32^ showed that coccolith calcite mass estimates calculated using birefringence-based methods were lower than morphometric-based approaches. They explain that the coccolith calcite mass of these species is systematically underestimated by birefringence-based methods because their coccolith thickness exceeds the theoretical thickness limit of 1.34 µm^32^. We are not aware of other studies where multiple methodologies for coccolith mass estimates have been directly compared on the same material for species with a broad range of coccolith thicknesses. Morphometric-based estimates of both coccolith and coccosphere calcite mass have also been shown to agree well with coccolith and coccosphere calcite mass values determined through opto-electrochemical dissolution^34,35^.

### Quantifying sources of uncertainty in morphometric-based estimates of cellular calcite

The morphometric-based calcite estimation method used here^30^ incorporates a number of (necessary) assumptions and sources of uncertainty. We summarise relevant caveats that have been raised previously in the literature^30^ and, based on our own observations, highlight additional methodological points that users of the dataset should be aware of:

- We use species- or genus-specific *Ks* values based on published *Ks* values where available. If no published *Ks* for a species or genus are available, we have either adopted the existing *Ks* value of a species with a very similar morphology or adjusted the *Ks* value of a species with similar morphology to account for the presence, absence or different degree of morphological features that are likely to increase or decrease the original *Ks* value. The value, source and justification for all *Ks* values used to estimate coccolith PIC in the database are detailed in Supplementary Table 1.
- Within-species, *Ks* values derived from individual coccolith measurements vary by ca. ±20%^30^. We note that the species-specific shape factors recommended by Young and Ziveri^30^ are based on measurements from a small number of individual coccoliths that are therefore unlikely to fully represent the range of shape factor variability within each species. For example, a study focusing on *E. huxleyi*^72^ showed strain-specific variability in *Ks*, ranging between 0.014 and 0.022 across seven morphotype A strains. A subsequent study on natural *E. huxleyi* populations^71^ emphasised that within-morphotype variability in coccolith mass can be substantial and that changes in coccolith size and coccolith thickness can be decoupled.
- Here, we assume that within-species coccolith shape and thickness remains constant even if coccolith size changes, although this is not always the case^72^. Coccolith thickness (and therefore mass) can vary independently of coccolith length and thickness variability can also be restricted to certain areas of the coccolith, such as changes in central tube cycle thickness^71,101,102^ or number and thickness of coccolith elements. As variation in coccolith size has a much stronger influence on coccolith volume than coccolith shape^30^, we did not account for shape variability with size in our coccosphere calcite calculations. It was also not possible to quantify variability in coccolith thickness across individual coccospheres in our samples and use this information to further refine our estimates coccolith calcite mass because methods to determine coccolith thickness are based on the birefringence of calcite in LM and our data is collected from SEM images.
- Within a species, coccolith size can vary considerably as a function of growth environment and genotypic variability. Even within a single genetic clone, coccolith size can vary by ca. 40% across individual cells as a function of variable environment and growth phase^4,69^. Many studies estimate species calcite using a mean species-specific coccolith length value. As we measure coccosphere-specific *C*_*L*_, we circumvent uncertainty that would be introduced by within-species size variability when using a mean *C*_*L*_ for all individual coccospheres of that species.
- A more relevant source of uncertainty for our measurements is that coccolith size (or spine length or other morphometric parameters used for calcite calculation) on a single coccosphere can also vary by up to ca. 30%^85,103^. This may occur through several mechanisms^104^: (1) cell size changes through the daily cell division cycle, from smallest immediately following cell division to largest just prior to cell division^103^ and during G1 interphase of the cell division cycle^105,106^. As heterococcolith coccolithogensis occurs intracellularly^107–109^, cell size at the time of coccolith formation can limit maximum coccolith dimensions. Coccoliths formed early in the daily cell cycle would therefore be likely to be slightly smaller than those formed later on; (2) As coccolith size is responsive to environmental stressors^22,52,110–116^, coccoliths produced under stressful conditions may have different sizes from those produced before exposure to the stressful condition. As approximately half of the coccoliths forming the coccosphere of the parent cell are divided equally amongst two daughter cells on cell division, cells will inherit a diminishing proportion of coccoliths of a different size originally from the parent cell (grown under different environmental conditions) over successive cell division cycles. It may therefore take several generations of coccolith production and growth before the coccosphere is composed of solely post-exposure coccoliths; (3) Small differences in the duration of coccolithogenesis by the cell could feasibly result in slight differences in coccolith size; (4) Large coccolith size variability on the same coccosphere is also a taxonomic feature of several species, which are highlighted in the genus descriptions in the Methods. We measure a single, approximately flat-lying coccolith on the upper surface of the coccosphere that we assume is representative of the size of all other coccoliths forming the coccosphere. However, it is likely that some coccoliths in the coccosphere are slightly smaller (and therefore have less calcite) and others are slightly larger (and therefore have more calcite). This is one of the larger sources of uncertainty in our calculations of coccosphere calcite from a single coccolith measurement (see below). For example, Beuvier *et al*.^103^ determined a threefold range in coccolith calcite (7–23 pg) within a coccosphere of *Gephyrocapsa oceanica* due largely to differences in coccolith size of 20–30%. A large range in coccolith size within the same coccosphere is a morphological feature of *Umbellosphaera* (Fig. 1m) and our measurements on collapsed coccospheres reveal that the difference in size between the smallest and largest coccoliths forming the coccosphere ranged from as little as 2.43 µm to as much as 8.27 µm, leading to a range in coccolith calcite within the same coccosphere of 2.4–28.4 pg coccolith^−1^ in *Umbellosphaera*^53^. For many species, the arrangement of coccoliths in the coccosphere rarely offers multiple, flat-lying coccoliths on the upper surface of the coccosphere for measurement (where the edge of the coccolith is not obscured by other coccoliths) or the *C*_*N*_ is high to very high, making it unreasonable to measure a representative selection of coccoliths on each coccosphere. As coccolith size variation is particularly notable in *Umbellosphaera*, we have specifically accommodated intra-coccosphere variation in *C*_*L*_ in our estimates of cellular calcite, which is described in the Methods.

**Fig. 3:**
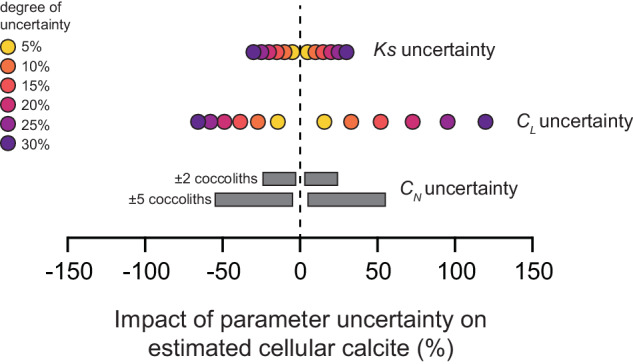
Sensitivity of estimated cellular calcite content to imposed uncertainties in calculation parameters. We re-calculated the cellular calcite content of four coccolithophore species (*Discosphaera tubifera* n = 20; *Syracosphaera* spp. n = 55; *Gephyrocapsa mullerae* n = 30; *Umbilicosphaera hulburtiana* n = 30) using a range of uncertainties in the parameters used in the calculation of cellular calcite: selected species-specific shape factor (Ks), measured coccolith length (*C*_*L*_), and number of coccoliths per coccosphere (*C*_*N*_). *Ks* and *C*_*L*_ were varied by ±5%, ±10%, ±15%, ±20%, ±25% and ±30%. *C*_*N*_ was varied by ±2 and ±5 coccoliths per coccosphere.

We investigated the sensitivity of cellular calcite estimated using morphometrics (Eqs. 4 and 5) to variability in coccolith size, number of coccoliths per cell, and *Ks* value to better quantify the magnitude of uncertainty introduced by the (necessary) assumptions made in our methodology (Fig. 3). For coccospheres of *Gephyrocapsa mullerae*, *Umbilicosphaera hulburtiana*, *Syracosphaera* spp., and *Discosphaera tubifera* (representing a selection of different morphological types and degrees of calcification), we re-calculated cellular calcite content by systematically varying *Ks* and *C*_*L*_ by ±5–30%. These ranges of variability are guided by the range of *Ks* reported by Young and Ziveri^30^ and Linge Johnsen *et al*.^72^ for *E. huxleyi* and the range of *C*_*L*_ on individual coccospheres reported for *Gephyrocapsa*^103^. To assess sensitivity to uncertainty in *C*_*N*_ counts, we varied *C*_*N*_ of each coccosphere by ±2 coccoliths and ±5 coccoliths.

Uncertainty in coccolith size (i.e., the measured coccolith is not representative of other coccoliths in the coccosphere) has by far the largest impact in estimated cellular calcite, irrespective of species, altering estimated cellular calcite by a minimum of ca. ±15% (for 5% uncertainty in *C*_*L*_) and decreasing cellular calcite content by a maximum of 65% or increasing it by a maximum of 120% for a 30% uncertainty in *C*_*L*_ (Fig. 3). The large impact of *C*_*L*_ uncertainty on estimated cellular calcite content arises because *C*_*L*_ is cubed in the calculation of coccolith calcite (Eq. 4). We consider a *C*_*L*_ uncertainty of 30%, whilst possible, to be an unreasonably large uncertainty that would only occur if the largest or smallest coccolith out of all the coccoliths on the coccosphere was measured^103^. A more reasonable level of uncertainty in measured *C*_*L*_ of ±10–15% between coccoliths on the same coccosphere results in estimated cellular calcite content decreasing/increasing by ca. 30% (10% *C*_*L*_ uncertainty) to decreasing by 39% or increasing by 52% (15% *C*_*L*_ uncertainty). Variability of *Ks* across individual coccoliths of, for example, ±20%^30^ results in cellular calcite estimates directly changing by ±20%. Errors in counting the number of coccoliths per cell can occur, and we estimated these to be ca. ±1–5 coccoliths per cell at most for the majority of coccospheres observed using SEM but conservatively as high as 10–40 coccoliths per cell counting error in species like the polycrater phase of *Alisphaera* that have several hundred very small coccoliths (Fig. 1f). Coccolith counting errors affect estimated cellular calcite proportionally to both the number of coccoliths per cell (i.e., the relative percentage counting error) and coccolith size (i.e., the calcite mass of each miscounted coccolith), thus giving a range of sensitivity in our analysis (Fig. 3). For the four species investigated, *C*_*N*_ ±2 resulted in a cellular calcite uncertainty of 2–22% and *C*_*N*_ ±5 resulted in a cellular calcite uncertainty of 5–55% for coccospheres with *C*_*N*_ ranging between 9 and 96. *C*_*N*_ counting errors are least likely in species with lower *C*_*N*_ and/or larger *C*_*L*_ and increase in likelihood in species whose coccospheres are comprised of very high numbers of coccoliths, although the typically small *C*_*L*_ of these higher-*C*_*N*_ species acts to minimise the overall impact of counting errors on cellular calcite estimations. Overall, it is reasonable to assume that slight differences in *Ks* and *C*_*L*_ between coccoliths on the same coccosphere and minor *C*_*N*_ counting errors introduces an uncertainty of ca. 5–40% on estimated cellular calcite.

### Quantifying sources of uncertainty in estimation of cell size from coccosphere size

The percentage of coccosphere volume that is cell volume (*y*) determined through LM varies between individual coccospheres^29,57,69,117^ but the intraspecific range in *y* is overall relatively low for many species. Based on 2850 measurements of coccosphere diameter and cell diameter (LM-based) for cultured *Calcidiscus* and *Helicosphaera*^69^ and *Coccolithus* plankton samples^68,117^, the percentage of coccosphere volume that was cell volume was relatively consistent across hundreds of individual coccospheres, varying by only 12–22% (10^th^ to 90^th^ percentiles of the data; cellular *y* for *Helicosphaera* = mean 51% ranging between 45% and 58%, cellular *y* for *Calcidiscus* = mean 42% ranging between 32% and 52%, and cellular *y* for *Coccolithus* = mean 45% ranging between 37% and 52%). As these species all have ‘heavily calcified’ coccolith morphologies (i.e., coccoliths are comparatively thick and solid structures) and coccospheres with strongly overlapping coccolith arrangements, we estimate that the variability in *y* in species with thinner coccoliths and those that have a lesser degree of or no overlapping coccolith arrangement (e.g., species bearing murolith- and planolith-type coccolith morphologies) is less than *y* ±10–15%. The relationship between coccosphere volume and cell volume is much more variable in *Florisphaera profunda* (up to *y* ±80%), *Umbellosphaera* spp. (up to *y* ±75%), *Oolithotus antillarum* (up to *y* ±37%), and *Acanthoica* spp. (up to *y* ±63%). These species have staggered, overlapping coccoliths arrangements that produce a very thick coccosphere relative to size of the cell and where additional (pseudo-)layers can be added with no change in cell size. The value of *y* for *Algirosphaera robusta* (up to *y* ±60%) depends on the variability in the height of central area processes, the morphology of which makes it impossible to measure coccosphere diameter with and without the height of the processes. Additionally, coccospheres of *Umbilicosphaera sibogae* contain multiple cells within a single coccosphere, making it extremely challenging to quantify *y* and its variability. For these specific taxa, the use of a fixed value for *y* was unavoidable but introduces uncertainty of ca. 30–60% in cell size estimates from coccosphere volume.

## Usage Notes

This dataset^53^ is generated from 16 plankton samples obtained from the temperate to equatorial Atlantic Ocean during 2004 and is therefore particularly well-suited for addressing research questions that focus on this region. As environmental conditions influence cell physiology, phenotype expression, biogeography and species’ evolution, coccolithophore populations present in different ocean basins, latitudes and/or hydrographic regimes may exhibit different ranges of morphological traits^43,82,115,118,119^ and species’ morphological traits have been shown to vary on a range of short-term (daily to seasonal) to longer-term (multi-annual to millennial) timescales^4,102,120–126^. As such, users of the dataset should note that the range of morphometric traits in the dataset are not necessarily representative of the full range of morphologies exhibited by each species globally and that the range of morphological traits present at specific temporal or spatial scales are dependent on the diversity of ecotypes present in the population. This may especially be true for species represented by a limited number of entries in the dataset (Fig. 2; Supplementary Table 1). A simple indicator of the range of morphometric data observed in the dataset relative to published literature is reported in Supplementary Table 1 for initial context.

Coccolithophore taxonomy is under constant refinement through morphological and, increasingly, genomic studies. We therefore strongly encourage users of the dataset to confirm the most up to date taxonomic identifier of each species in the dataset before use, for example using the Nannotax3 website^23^ as this resource is constantly maintained, provides details of previous species synonyms, and provides extensive references to primary literature.

Users of the dataset may wish to use one of the several published cell volume to cell biomass relationships^29,67,127^ to further convert the cell size data in the dataset into estimates of cellular organic carbon (biomass).

Identical morphometric measurements to those presented here can also be made on intact fossil coccospheres preserved in marine sediments^66,68,128,129^. Using the same morphometric-based method^30^, the cellular calcite of extinct calcareous nannoplankton species can be estimated and used to investigate evolution in cellular calcite content through time^31,66,68,130^.

### Supplementary information

Supplementary Table 1

## Data Availability

No custom code was used for the generation or processing of the datasets used in this study.
