# Supplementary material for: Cellular morphological trait dataset for extant coccolithophores from the Atlantic Ocean
Source: Sci Data. 2024 Jul 2;11:720. doi: 10.1038/s41597-024-03544-1 (PMC11220069; doi:10.1038/s41597-024-03544-1)
Supplement: Supplementary file 1 — Supplementary Table 1 [file 41597_2024_3544_MOESM1_ESM.pdf]

Supplementary Information for Sheward et al. "*Cellular morphological trait dataset for extant coccolithophores from the Atlantic Ocean*"

Supplementary Table 1: Summary of species- and genus-specific morphological characteristics for extant coccolithophore species or genera reported in the dataset and used to estimate cellular calcite. All morphometric data is measured on individual intact coccospheres observed using scanning electron microscopy, imaged from samples from the Atlantic Meridional (AMT) 14 cruise in 2004. See Table 1 for details. n= number of intact coccospheres in dataset. C_L = coccolith length (µm) of a representative coccolith on each coccosphere. *Ks* = taxon-specific shape factor used to estimate coccolith volume as a function of coccolith morphology and coccolith thickness. ∅ = coccosphere (equivalent spherical) diameter (µm). *y* = taxon-specific percentage coccosphere volume that is cell volume (based on light microscopy measurements) used for converting measured coccosphere size into an estimate cell size. Note that there are two stated ranges for morphological parameters (C_L, ∅, C_N): data range A refers to the range within our dataset whilst data range B refers to ranges reported in the literature as summarised on Nannotax3. Discrepancies between the range of data in our dataset and in the literature arise because our dataset may not represent the full range of morphologies observed across each species globally and under all environmental conditions, as our measurements are from extant Atlantic Ocean populations from a single cruise only. Coccolith lith type and shape: BC= body coccolith, C=circular, E=elliptical, A=asymmetrical, Q=quadrate, R=rod-shaped. Coccosphere shapes: E=elliptical, PS=prolate spheroid (sub-spherical), S=spherical, T=tubular (C_L>2x*coccolith width*), DC=double cone. See volume calculations in the Methods based on Sun and Liu (2003). Other features: CFCs = circum-flagellar coccoliths (coccoliths of distinct morphology occurring around the flagellar opening), AACs = antapical coccoliths (coccoliths of distinct morphology occurring at the antapical pole), XCs = exothecal coccoliths (coccoliths of distinct morphology occurring in the outer layer of two-layer coccospheres).

		**Coccolith morphological traits**							**Coccosphere morphological traits**					
**Species/genus**	**n**	**BC lith type**	**BC shape**	**Central area**	**Dimension for calcite calculation**	**C_L range (µm)**	**Ks**	**Source of Ks value**	**Shape**	**∅ range (µm)**	***y***	**C_N range**	**Multilayer or canopy structure**	**Other features**
Acanthoica spp.	11	Planolith Polymorphic, some coccoliths have well-developed spines	C to E	Grill, processes	length	A: 1.5-2.6<br>B: 1-4	0.03	Young and Ziveri (2000)	S to PS	A: 6-8<br>B: 6-12	56%	A: 28-90<br>B: 25-105	No	CFCs, AACs
Algirosphaera robusta	5	Planolith	E	Grill, processes	length	A: 2.0-3.4<br>B: 1.5-4.5	0.045	Daniels et al. 2014	S	A: 10-14<br>B: 6.5-16	32%	A: 30-130<br>B: 70-90	No	-
Alisphaera spp.	4	Placolith	E, A	-	length	A: 1.0-1.4<br>B: 1-3	0.04	Placolith with very narrow central area. Estimated as higher than *Syracosphaera* and *Acanthoica*	S	A: 5.5-8.5<br>B:4.5-16	65%	A: 56-80<br>B: 80-150 for *A. unicornis*	No	-
Alisphaeraceae polycrater phase	2	Nannolith	Q	-	length	A: 0.5-0.6<br>B: 0.3-1<br>Fixed C_L = 0.8 when necessary	0.01	Arbitrary small *Ks* value used	S	A: 7-12<br>B: 5-16	76%	(200-1300)<br>Fixed C_N =400 when necessary	No	-
Calcidiscus spp.	18	Placolith	C	Closed	length	A: 4.7-7.8<br>B: 3-10	0.08	Young and Ziveri (2000)	S	A:11-17.5<br>B: 5-20	41%	A:20-48<br>B:20-50	No	-
Calciosolenia spp.	13	Murolith	Q	Grill	length	A: 1.8-2.9<br>B: 2.5-7	0.007	Back-calculation of *Ks* using coccolith calcite (2.5 pg) and length (5 µm) data from Bollmann (2014)	DC	A: 3-7<br>B: 20-95 (refers to coccosphere long axis)	90%	A: 40-106<br>B: 80-250	No	AACs, CFCs in *C. murrayi*
Ceratolithus vidalii HET (previously *nishiidae)*	1	Planolith	C	Open	length	A:4.6<br>B: 4-7	0.03	*Ceratolithus* heterococcoliths with narrow central opening so assume double *C. cristatus* HET, similar to *Acanthoica*	S	A: 8.6<br>B: 7-12	32%	A: 20<br>B: 10-15	No	-
Discosphaera tubifera	20	Planolith	E	Grill, processes (trumpet-like)	Trumpet width	A: 1.2-3.6<br>B: 3-8	0.07	Young and Ziveri (2000)	S	A: 3-9 (excluding process height)<br>B: 12-20 (including process height)	95% of size excl. trumpet height	A: 13-96<br>B: 29-70	No	-

Supplementary Table 1 continued:

		Coccolith morphological traits							Coccosphere morphological traits					
**Species/genus**	**n**	**BC lith type**	**BC shape**	**Central area**	**Dimension for calcite calculation**	**C_L range (µm)**	**Ks**	**Source of Ks value**	**Shape**	**∅ range**	***y***	**C_N range**	**Multilayer or canopy structure**	**Other features**
Emiliania huxleyi	338	Placolith	E	Grill, open or closed depending on morphotype	length	A: 1.7-4<br>B: 2-5	0.02	Young and Ziveri (2000). We do not account for different morphotypes.	S	A: 3-11.6<br>B: 4-10	86%	A: 5-82<br>B: 10-50	Common	-
Florisphaera profunda	32	Nannolith	Q, R	-	length	A: 1.2-3.8<br>B: 1.5-6	0.03	Linge Johnsen and Bollmann (2020)	S to E	A: 4.8-12<br>B: 6-16	10%	A: fixed at 145<br>B: 92-165	artichoke-shaped coccospheres of overlapping layers of liths	-
Gephyrocapsa spp.	188	Placolith	E	Grill, bar	length	A: 1-4<br>B: 1-6	0.05	Young and Ziveri (2000)	S	A: 2.5-6.2<br>B: 3-10	30%	A: 6-24<br>B: 9-35	No	-
Helicosphaera spp.	12	Placolith	A, E	Bar or closed	length	A: 4.7-9.4<br>B: 4-12	0.05	Young and Ziveri (2000)	PS to E	A: 9.8-21.6<br>B: 9-25	51%	A: 11-66<br>B: 15-50	No	-
Holococcolithophore species	65	Holococcolith	Varied	Varied	length	Varied	0.036	Daniels et al. (2014)	varied	Varied	80%	Varied	No	-
Michaelsarsia elegans	5	Murolith	E	Grill, plate, processes	Length (BCs)	A: 1.9-2.5<br>B: 1.8-2.5	0.02	Adapted from *Syracosphaera*	E	A: 7-9<br>B: 10-20	67%	A: 40-60<br>B: 45-150	No	ACCs, CFCs, XCs, appendages
Oolithotus spp.	34	Placolith	A, C	Closed	length	A:2.7-5.9<br>B: 3-9	0.07	Young and Ziveri (2000)	E, A	A: 4.7-15<br>B: 6-20	40%	A: 11-44<br>B: 15-60	No	-
Ophiaster spp.	8	Murolith	E	Grill, process	length	A: 1-1.6<br>B: 0.7-1.8	0.015	Adapted from *Syracosphaera*, similar BC morphology	S to E	A: 5.3-9.3<br>B: 4-8	67%	A: 34-84<br>B: 50-100	No	Appendages
Rhabdosphaera clavigera var. *clavigera*	12	Planolith<br>BC dimorphic (with or without spines)	E	Process	Spine length, length	A: 4-5 spine length, 2.5-3.7 base length<br>B: 7-10 spine length, 3-3.5 base length	0.025	Young and Ziveri (2000)	S	A: 5.8-7.7 excl. processes<br>B: 20-35 incl. processes, 8.5-10.5 excl. processes	85%	A: 20-58<br>B: 22-52	No	Base length and spine length measured
Rhabdosphaera clavigera var. *stylifera*	6	Planolith<br>BCs dimorphic (with or without spines)	E	Process	Spine length, length	A: 3-3.8, 4-7.5 spine length<br>B: 3-5 spine length	0.015	Young and Ziveri (2000)	S	A: 6.3-9.9 excl. processes<br>B: 8-15 incl. processes, 4.5-7.5 excl. processes	85%	A: 26-56<br>B: 30-40	No	Base length and spine length measured
Syracosphaera<br>- *borealis* type<br>- *nodosa* type<br>- *Syracosphaera maxima*	27	Murolith<br>This group is very diverse, many species polymorphic	C to E	Variable	length	A: 1.3-3.2<br>B: 0.9-3.5	0.015	Young and Ziveri (2000)	S to E	A: 4.7-10.8<br>B: 3-13	75%	A: 19-80	Dithecal coccospheres common (BCs and XCs)	Often XCs
Syracosphaera<br>- *molischii* gr. (excl. *borealis* type)	70	Placolith-like	E	No spines, often with ridges	length	A: 1-3.5<br>B: 1-3.5	0.022	Arbitrary intermediate *Ks* value between more heavily calcified *Syracosphaera* morphologies and less heavily calcified *Syracosphaera* morphologies	S to PS	A: 2.8-17.9<br>B: 3-20	75%	A: 14-92<br>B: 20-70	Dithecal coccospheres common (BCs and XCs)	Often XCs, CFCs with spines in '*molischii* type'

Supplementary Table 1 continued:

		Coccolith morphological traits							Coccosphere morphological traits					
**Species/genus**	**n**	**BC lith type**	**BC shape**	**Central area**	**Dimension for calcite calculation**	**C_L range (µm)**	**Ks**	**Source of Ks value**	**Shape**	**Ø range**	***y***	**C_N range**	**Multilayer or canopy structure**	**Other features**
Syracosphaera - *pulchra* gr. - *Syracosphaera mediterranea*	20	Murolith Polymorphic BCs with flange CFCs similar with spine XCs dome-shaped	E	Grill, process	length	A: 1.3-6 B: 1.1-8	0.03	Young and Ziveri (2000)	E to T	A: 3.2-19.9 B: 8-70 (length of *S. prolongata*)	65%	A: 20-100 B: 20-100	Dithecal coccospheres common (BCs and XCs)	CFCs, XCs
Tetralithoides quadrilaminata	5	Placolith	E	Plate, closed	length	A: 4-5 B: 4-7.5	0.015	Adapted from *Syracosphaera*, small	S	A: 19-21.6 B: 15-25	94%	A: 80-104 B: 70-130	No	-
Umbellosphaera irregularis	36	Placolith Size is varimorphic coccospheres)	E, A	Plate, closed	Trumpet width	A: 2-10.4 B: 1.4-10 'trumpet' length	0.01	Young and Ziveri (2000)	S	A: 6-14.6 B: 10-15 incl. trumpet processes	13%	A: 8-42 B: 14-30	Canopy effect of overlapping coccoliths can obscure smaller, underlying coccoliths.	-
Umbellosphaera tenuis	88	Placolith Size is strongly varimorphic	E, A	Plate, closed	Trumpet width	A: 3.4-7.1 B: 2-9 'trumpet length	0.015	Young and Ziveri (2000)	S	A: 5.7-11 B: 8-12 incl. trumpet processes	19%	A: 12-39 B: 15-25	Canopy effect of overlapping coccoliths obscures smaller, underlying coccoliths.	-
Umbilicosphaera hulburtiana	49	Placolith	E	Open	length	A: 3.2-5.1 B: 4-6	0.055	Adapted from *U. foliosa* and *Coccolithus*	S	A: 7.9-11.9 B: 8-10	40%	A: 18-54 B: 14-30	No	-
Umbilicosphaera sibogae	3	Placolith	C	Open	length	A: 4.5-7 B: 3-6	0.05	Young and Ziveri (2000)	S	A: 14.5-26.8 B: 20-30	90% (accounts for the inner coccosphere size but in this species that does not equate to cell size as coccospheres contain multiple cells)	A: 34-176 B: 40-200	No	Coccospheres usually contain 2-4 cells

References

Bollmann, J. Technical Note: Weight approximation of coccoliths using a circular polarizer and interference colour derived retardation estimates - (The CPR Method). *Biogeosciences* **11**, 1899–1910 (2014).

Daniels, C. J., Tyrrell, T., Poulton, A. J. & Young, J. R. A mixed life-cycle stage bloom of Syracosphaera bannockii (Borsetti and Cati, 1976) Cros et al . 2000 (Bay of Biscay, April 2010). *J. Nannoplankt. Res.* **34**, 31–35 (2014)

Linge Johnsen, S. A. & Bollmann, J. Segmentation, retardation and mass approximation of birefringent particles on a standard light microscope. *J Microsc* **280**, 30-50 (2020).

Sun, J. & Liu, D. Geometric models for calculating cell biovolume and surface area for phytoplankton. *J. Plankton Res.* **25**, 1331–1346 (2003)

Young, J. R. & Ziveri, P. Calculation of coccolith volume and it use in calibration of carbonate flux estimates. *Deep-Sea Res Pt II* **47**, 1679–1700 (2000).
